# Mortality of Philadelphia, for July, August and September, 1853, Collated from the Record Kept at the Health Office

**Published:** 1853-11

**Authors:** Wilson Jewell


### MEDICAL EXAMINER,

AND

### RECORD OF MEDICAL SCIENCE.

NEW SERIES.—NO. CVII.—NOVEMBER, 1853.

### ORIGINAL COMMUNICATIONS.

*Mortality of Philadelphia, for July, August and September, 1853, collated from the record kept at the Health Office.* By Wilson Jewell, M. D.

The total of deaths for the quarter ending October 1st, 1853, a period of 91 days or 13 weeks, amounts to 2955, equal to $32\frac{1}{2}$ deaths per diem. An increase over the same quarter in 1852 of 204 in the aggregate, or about 7 per cent.

If we deduct the Still Born 137, the deaths from External Causes 124, Old Age 30, Debility 116, and Unknown 60, it gives us a record of 2488 deaths from reported diseases. Nevertheless, it will be borne in mind, that this is a mere approximation to the truth, owing in a great measure to the too frequent disregard by many practitioners, of the proper investigation of disease, and the cause of death and to the *ad libitum* manner of certification.

The excess of deaths in the sexes has been, as usual, on the side of males; equal to ten per cent.

Thirteen hundred and seventy-two, or nearly one-half the whole number of deaths, exclusive of Still Born, were under five years of age.

The record during the present quarter shows a considerable increase of mortality over that of the two previous quarters, amounting to 16 per cent, above the first, and 25 per cent, above the second quarter.

This augmentation, notwithstanding the absence from the city of many of our citizens, is the result generally of infantile mortality, during the *summer solstice.* But the present quarter's increase has been somewhat enhanced from the more than usual prevalence of Fever, in a certain locality of the city.

CLASS 1st.—Embracing the "Zymotic or Epidemic" diseases, furnishes 999 of the deaths for the quarter. Of this number, Cholera Infantum supplies 308, Dysentery 248, and Fevers of every division 266. Among the Fevers, 134 are set down under the head of those usually ascribed to malarious causes. This is a marked excess above those for the same period in 1852, when only 26 deaths were recorded from a similar cause. Last year there was an entire exemption from any Zymotic influence. The present quarter's unfair comparison in this respect, with the figures of other years, must be attributed in no small degree to the existence of an unusual poison in the atmosphere.

This poison has been productive of a highly malignant type of Fever, which not only assumed the livery of, but was in fact Yellow Fever itself. Of these, only 24 are recorded as Yellow Fever, while the remainder are put down under various other heads, as Typhus Icterodes, Malignant, &c.

It is proper to observe, that while the cases of Fever have increased in an undue proportion, over those for the same quarter in 1852, the comparative aggregate of deaths in the first class for the present quarter has only exceeded those of 1852, nineteen!

CLASS 2d.—"Sporadic." Of these, there were 400 deaths. Nearly one half occurred within the first year of life. Among them, 91 were from Marasmus, 48 from Debility and 22 from Inanition.

CLASS 3d.—"Nervous System." In this division, convulsions, that formidable cause of mortality in children, are credited with 165, out of the 509 deaths enumerated. The comparative increase of deaths from "sun stroke," (numbering 33, which, added to those for the second quarter, 24, making in all 57,) above those for the year 1852, which only numbered 5, is an evidence of the high thermometrical atmosphere under which we lived during the summer.

Class 4th.—"Organs of Respiration." Under this general distinction, we find the deaths from consumption of the lungs amounting to 282, an increase over those of the same period for 1852, of 30, or nearly eleven per cent.

Table No. 3 exhibits the number of death at fifteen separate periods of life. Here we find that infancy supplies a very liberal proportion, not less than 1,372 occurring before the fifth year. Between the ages of twenty and fifty there were 833 deaths, showing that these distinct periods of infancy and manhood are fruitful in the production of death.

The deaths from the Alms House numbered 221. Those among the blacks 175, while there were 35 brought from the country to swell our city mortality.

TABLE No. I.

*Deaths for the Third Quarter of 1853, classified.*

| | July. | August. | September. | MALE. | | | FEMALE. | | | Total. |
|---|---|---|---|---|---|---|---|---|---|---|
| | | | | J. | A. | S. | J. | A. | S. | |
| 1. *Endemic and Contagious Diseases.* Zymotic or Epidemic | 314 | 446 | 239 | 169 | 249 | 118 | 145 | 197 | 121 | 999 |
| 2. *Uncertain or general seat.* Sporadic diseases | 134 | 149 | 117 | 75 | 72 | 61 | 59 | 77 | 56 | 400 |
| 3. Nervous System | 153 | 231 | 125 | 84 | 146 | 72 | 69 | 85 | 53 | 509 |
| 4. Organs of Respiration | 115 | 157 | 145 | 55 | 67 | 65 | 60 | 90 | 80 | 417 |
| 5. " Circulation | 12 | 19 | 13 | 3 | 8 | 7 | 9 | 11 | 6 | 44 |
| 6. Digestive Organs | 64 | 79 | 38 | 30 | 42 | 22 | 34 | 37 | 16 | 181 |
| 7. Urinary Organs | 4 | 4 | 1 | 1 | 4 | | 3 | | 1 | 9 |
| 8. Organs of Generation | 9 | 10 | 13 | | | | 9 | 10 | 13 | 32 |
| 9. " Locomotion | 1 | 3 | 1 | 1 | 3 | | | | 1 | 5 |
| 10. Integumentary System | 3 | 1 | 4 | 2 | 1 | 3 | 1 | | 1 | 8 |
| 11. Old Age | 11 | 9 | 10 | 6 | 2 | | 5 | 7 | 10 | 30 |
| 12. External Causes | 37 | 56 | 31 | 26 | 42 | 19 | 12 | 14 | 11 | 124 |
| Still Born | 44 | 47 | 46 | 21 | 23 | 22 | 23 | 24 | 24 | 137 |
| Unknown | 19 | 24 | 17 | 9 | 15 | 13 | 10 | 9 | 4 | 60 |
| | 920 | 1235 | 800 | 482 | 674 | 402 | 439 | 561 | 397 | 2955 |

TABLE No. II.

1. *Endemic and Contagious Diseases—Zymotic or Epidemic.*

| | Male. | Female. | Under 1 yr. | 1 to 2. | 2 to 5. | 5 to 10. | 10 to 15. | 15 to 20. | 20 to 30. | 30 to 40. | 40 to 50. | 50 to 60. | 60 to 70. | 70 to 80. | 80 to 90. | 90 to 100. | 100 to 110. | Total. |
|---|---|---|---|---|---|---|---|---|---|---|---|---|---|---|---|---|---|---|
| Cholera Asphyxia . | 2 | | | | | | | | 1 | 1 | | | | | | | | 2 |
| " Infantum . | 162 | 146 | 216 | 85 | 7 | | | | | | | | | | | | | 308 |
| " Morbus . | 10 | 4 | 3 | | | 1 | | | | 4 | 4 | | | 2 | | | | 14 |
| Croup . | 19 | 12 | 4 | 5 | 18 | 4 | | | | | | | | | | | | 31 |
| Diarrhœa . | 38 | 33 | 26 | 14 | 8 | 2 | 1 | 1 | 1 | 3 | 4 | 4 | 5 | | 2 | | | 71 |
| Dysentery . | 142 | 106 | 37 | 49 | 22 | 17 | 9 | 8 | 24 | 16 | 28 | 18 | 16 | 4 | | | | 248 |
| Erysipelas . | 3 | 5 | | | 1 | | | | 3 | 4 | | | | | | | | 8 |
| Fever . | 3 | 4 | 2 | | | | | 1 | 2 | | 2 | | | | | | | 7 |
| " Bilious . | 17 | 10 | | | 1 | | | 1 | 8 | 5 | 2 | 2 | 5 | 3 | | | | 27 |
| " Congestive . | 1 | 3 | | | | | | 1 | 1 | 1 | | 1 | | | | | | 4 |
| " Continued . | | 1 | | | 1 | | | | | | | | | | | | | 1 |
| " Intermittent . | 3 | 2 | | | 1 | 1 | | | | | 1 | 1 | | 1 | | | | 5 |
| " Malignant . | 8 | 7 | | | | | 1 | | 4 | 2 | 6 | 2 | | | | | | 15 |
| " " Bilious . | 12 | 17 | | | | | | 3 | 13 | 5 | 6 | 1 | 1 | | | | | 29 |
| " " Remittent | 1 | 1 | | | | | | 1 | 1 | | | | | | | | | 2 |
| " Nervous . | | 1 | | | | | | 1 | | | | | | | | | | 1 |
| " Pernicious . | | 1 | | | | | | | 1 | | | | | | | | | 1 |
| " Remittent . | 10 | 5 | | 1 | '1 | 3 | 2 | 2 | 2 | 3 | | 1 | | | | | | 15 |
| " Scarlet . | 15 | 19 | 5 | 5 | 13 | 9 | | 2 | | | | | | | | | | 34 |
| " Typhus . | 16 | 14 | | | 1 | 1 | 1 | 6 | 5 | 7 | 4 | 4 | 1 | | | | | 30 |
| " Typhoid . | 31 | 29 | | | 1 | 1 | 4 | 10 | 17 | 9 | 10 | 3 | 3 | 2 | | | | 60 |
| " Typhus Icterodes | 9 | 2 | | | | | | 2 | 9 | | | | | | | | | 11 |
| " Yellow . | 11 | 13 | | | 1 | | | 2 | 12 | 6 | 3 | | | | | | | 24 |
| Hooping Cough . | 14 | 25 | 20 | 11 | 6 | 2 | | | | | | | | | | | | 39 |
| Measles . | 1 | | | 1 | | | | | | | | | | | | | | 1 |
| Small Pox . | 5 | 2 | 4 | | 1 | 2 | | | | | | | | | | | | 7 |
| Syphilis . | 2 | | | | 1 | | | | 1 | | | | | | | | | 2 |
| " Congenital . | 1 | 1 | 2 | | | | | | | | | | | | | | | 2 |
| | 536 | 463 | 319 | 171 | 84 | 43 | 18 | 41 | 105 | 66 | 70 | 37 | 31 | 12 | 2 | | | 999 |

2. *Uncertain or General Seat.—Sporadic Diseases.*

| | Male. | Female. | Under 1 yr. | 1 to 2. | 2 to 5. | 5 to 10. | 10 to 15. | 15 to 20. | 20 to 30. | 30 to 40. | 40 to 50. | 50 to 60. | 60 to 70. | 70 to 80. | 80 to 90. | 90 to 100. | 100 to 110. | Total. |
|---|---|---|---|---|---|---|---|---|---|---|---|---|---|---|---|---|---|---|
| Abscess . . . | 6 | 3 | 1 | | 2 | 1 | | | 1 | 1 | 2 | 1 | | | | | | 9 |
| " Psoas . . | 1 | | | | | | | | | | | 1 | | | | | | 1 |
| Cachexia . . . | | 2 | 1 | | | | | | | | | | | 1 | | | | 2 |
| Cancer . . . | 1 | 2 | | | | | | | | 1 | | | 1 | 1 | | | | 3 |
| " Breast . . | | 4 | | | | | | | | | 1 | 3 | | | | | | 4 |
| " Lip . . . | 1 | | | | | | | | 1 | | | | | | | | | 1 |
| Cyanosis . . . | 2 | 5 | 6 | 1 | | | | | | | | | | | | | | 7 |
| Debility . . . | 45 | 71 | 48 | 4 | 3 | | 1 | 1 | 5 | 8 | 4 | 8 | 18 | 10 | 4 | 2 | | 116 |
| Disease of Glands . . | 1 | | | | | | | | | | | 1 | | | | | | 1 |
| Dropsy . . . | 16 | 18 | 1 | | 2 | 3 | 1 | | 3 | 8 | 3 | 2 | 6 | 2 | 2 | 1 | | 34 |
| Disease of Abdomen . | 1 | | | | | | | | | 1 | | | | | | | | 1 |
| Hemorrhage . . | 3 | 1 | 1 | 1 | | | 1 | | | | | 1 | | | | | | 4 |
| Inanition . . . | 18 | 9 | 22 | 1 | | | | | 1 | | 1 | | | 2 | | | | 27 |
| nflammation . . | | 2 | | 1 | 1 | | | | | | | | | | | | | 2 |
| " Glands (malignant) | 1 | | | | | 1 | | | | | | | | | | | | 1 |
| Marasmus . . . | 85 | 60 | 91 | 40 | 10 | 1 | 1 | | | | | | | 1 | 1 | | | 145 |
| Malformation . . | 2 | 1 | 3 | | | | | | | | | | | | | | | 3 |
| Melanosis . . . | 1 | | | | | | | | | | 1 | | | | | | | 1 |
| Melœna . . . | | 1 | 1 | | | | | | | | | | | | | | | 1 |
| Mortification . . | | 1 | | | | | | | 1 | | | | | | | | | 1 |
| Tabes Mesenterica. . | 9 | 5 | 4 | 5 | 5 | | | | | | | | | | | | | 14 |
| Scrofula . . . | 11 | 5 | 1 | 1 | 4 | 2 | 1 | | 3 | 1 | 1 | 1 | 1 | | | | | 16 |
| Tumor, Abdominal . | | 2 | | | | | | | | | | | | 1 | 1 | | | 2 |
| Ulceration, Throat . | 4 | | 2 | | 2 | | | | | | | | | | | | | 4 |
| | 208 | 192 | 182 | 54 | 29 | 8 | 5 | 1 | 15 | 20 | 13 | 18 | 26 | 18 | 8 | 3 | | 400 |

3. *Of the Nervous System.*

| | Male. | Female. | Under 1 yr. | 1 to 2. | 2 to 5. | 5 to 10. | 10 to 15. | 15 to 20. | 20 to 30. | 30 to 40. | 40 to 50. | 50 to 60. | 60 to 70. | 70 to 80. | 80 to 90. | 90 to 100. | 100 to 110. | Total. |
|---|---|---|---|---|---|---|---|---|---|---|---|---|---|---|---|---|---|---|
| Abscess of Brain . . | 1 | 1 | | | 1 | | | | | | 1 | | | | | | | 2 |
| Apoplexy . . . | 20 | 12 | | | | | | | 2 | 9 | 5 | 3 | 7 | 5 | | 1 | | 32 |
| Concussion of Brain . | 1 | 1 | | | | | | | 1 | 1 | | | | | | | | 2 |
| Congestion " . | 49 | 21 | 15 | 9 | 9 | 2 | 1 | 4 | 5 | 13 | 1 | 7 | 3 | 1 | | | | 70 |
| Compression " . | 2 | 2 | | 1 | | | | | | 1 | 1 | | 1 | | | | | 4 |
| Convulsions . . | 81 | 75 | 88 | 39 | 18 | 4 | 2 | | 2 | 2 | | 1 | | | | | | 156 |
| Coup de Soleil . . | 25 | 8 | | | 1 | | | | 10 | 12 | 6 | 2 | 1 | 1 | | | | 33 |
| Dementia . . . | | 2 | | | | | | | 1 | | | | 1 | | | | | 2 |
| Disease of Brain . . | 12 | 13 | 7 | 9 | 2 | 2 | 1 | | 1 | 1 | 1 | | | | 1 | | | 25 |
| Dropsy of Brain . . | 31 | 24 | 23 | 14 | 13 | 2 | 1 | | 1 | 1 | | | | | | | | 55 |
| Effusion " . . | 13 | 11 | 8 | 2 | 6 | 2 | | | 2 | 2 | | 1 | 1 | | | | | 24 |
| Epilepsy . . . | 3 | 2 | 1 | | | 2 | | 1 | | | 1 | | | | | | | 5 |
| Inflammation of Brain . | 24 | 17 | 13 | 8 | 4 | 5 | 2 | 2 | 2 | 1 | 3 | | 1 | | | | | 41 |
| Mania . . . | 1 | | | | | | | | | | | | 1 | | | | | 1 |
| " a potu . . | 17 | 8 | | | | | | | 5 | 6 | 9 | 5 | | | | | | 25 |
| " Puerperal . . | | 1 | | | | | | | 1 | | | | | | | | | 1 |
| Neuralgia . . . | 1 | 1 | 1 | | | | | | 1 | | | | | | | | | 2 |
| Neurosis . . . | 1 | | | | | | | | 1 | | | | | | | | | 1 |
| Palsy . . . . | 13 | 7 | | | 1 | | | | 1 | 2 | 2 | 5 | 1 | 2 | 5 | 1 | | 20 |
| Softening of Brain . | 1 | | | | | | | | | | | | 1 | | | | | 1 |
| Tetanus . . . | 4 | | | | | | | 1 | 2 | 1 | | | | | | | | 4 |
| Trismus . . . | | 1 | | | | | | | | | | | 1 | | | | | 1 |
| Tumors of Brain . | 2 | | | | | | | | | | | | 1 | 1 | | | | 2 |
| | 302 | 207 | 156 | 82 | 55 | 19 | 7 | 8 | 38 | 52 | 30 | 24 | 20 | 10 | 6 | 2 | | 509 |

4. *Organs of Respiration.*

| | Male. | Female. | Under 1 yr. | 1 to 2. | 2 to 5. | 5 to 10. | 10 to 15. | 15 to 20. | 20 to 30. | 30 to 40. | 40 to 50. | 50 to 60. | 60 to 70. | 70 to 80. | 80 to 90. | 90 to 100. | 100 to 110. | Total. |
|---|---|---|---|---|---|---|---|---|---|---|---|---|---|---|---|---|---|---|
| Asthma . . . | 1 | 2 | | | | | | | | | | 1 | 1 | 1 | | | | 3 |
| Congestion of Lungs . | 6 | 5 | 1 | 1 | 1 | | | 1 | 2 | 1 | | 4 | | | | | | 11 |
| Consumption " . | 127 | 155 | 6 | 11 | 5 | 1 | 3 | 30 | 89 | 58 | 40 | 21 | 11 | 7 | | | | 282 |
| Disease of Chest . . | 1 | 1 | 1 | | | | | 1 | | | | | | | | | | 2 |
| " Lungs . | 2 | 6 | 2 | 1 | | | | 1 | 2 | 1 | 1 | | | | | | | 8 |
| Dropsy of Chest . . | 7 | 5 | | | 3 | | | | | 2 | | 6 | | 1 | | | | 12 |
| Effusion " . . | 1 | 1 | | | | | | | | | 1 | | | | 1 | | | 2 |
| Hemorrhage of Lungs . | 4 | 3 | | | | | | | 1 | 3 | 1 | 1 | 1 | | | | | 7 |
| Inflammation Bronchiæ . | 13 | 17 | 10 | 9 | 2 | | | | 1 | | 2 | | 2 | 3 | 1 | | | 30 |
| " Chest . | 2 | 1 | 2 | | | | | 1 | | | | | | | | | | 3 |
| " Larynx . | | 3 | | 1 | 1 | 1 | | | | | | | | | | | | 3 |
| " Lungs . | 20 | 27 | 14 | 4 | 7 | 4 | | | 7 | 6 | 3 | 1 | 1 | | | | | 47 |
| " Pleura . | 1 | | | | | | | 1 | | | | | | | | | | 1 |
| Hectic Fever . . | | 1 | 1 | | | | | | | | | | | | | | | 1 |
| Tuberculosis . . | | 3 | | | 1 | | | | 2 | | | | | | | | | 3 |
| Ulceration of Larynx . | 1 | | | | 1 | | | | | | | | | | | | | 1 |
| " Trachea . | 1 | | | | 1 | | | | | | | | | | | | | 1 |
| | 187 | 230 | 37 | 27 | 22 | 6 | 3 | 35 | 104 | 71 | 48 | 34 | 16 | 12 | 2 | | | 471 |

5. *Organs of Circulation.*

| | Male. | Female. | Under 1 yr. | 1 to 2. | 2 to 5. | 5 to 10. | 10 to 15. | 15 to 20. | 20 to 30. | 30 to 40. | 40 to 50. | 50 to 60. | 60 to 70. | 70 to 80. | 80 to 90. | 90 to 100. | 100 to 110. | Total. |
|---|---|---|---|---|---|---|---|---|---|---|---|---|---|---|---|---|---|---|
| Aneurism of Aorta . | 2 | | | | | | | | | 1 | 1 | | | | | | | 2 |
| Disease of Heart . . | 12 | 16 | 4 | | | | | | 6 | 7 | 2 | 2 | 4 | 2 | 1 | | | 28 |
| Enlargem't " . . | 2 | 5 | 1 | | | | 2 | | | | 2 | 1 | 1 | | | | | 7 |
| Inflammation " . . | | 2 | | | | | | | 1 | 1 | | | | | | | | 2 |
| Malformation " . . | 1 | 2 | 3 | | | | | | | | | | | | | | | 3 |
| Ossification " . . | 1 | | | | | | | | | 1 | | | | | | | | 1 |
| Syncope . . . | | 1 | | | | | | | 1 | | | | | | | | | 1 |
| | 18 | 26 | 8 | | | | 2 | | 8 | 10 | 5 | 3 | 5 | 2 | 1 | | | 44 |

### 6. *Digestive Organs.*

| | Male. | Female. | Under 1 yr. | 1 to 2. | 2 to 5. | 5 to 10. | 10 to 15. | 15 to 20. | 20 to 30. | 30 to 40. | 40 to 50. | 50 to 60. | 60 to 70. | 70 to 80. | 80 to 90. | 90 to 100. | 100 to 110. | Total. |
|---|---|---|---|---|---|---|---|---|---|---|---|---|---|---|---|---|---|---|
| Aphthæ . . . . | | 2 | 2 | | | | | | | | | | | | | | | 2 |
| Cancer of Mesent'c Glands | 1 | | | | | | | | | | 1 | | | | | | | 1 |
| Cancer of Stomach . | | 1 | | | | | | | | | | 1 | | | | | | 1 |
| Cancrum Oris . . | | 3 | 1 | 1 | 1 | | | | | | | | | | | | | 3 |
| Cirrhosis Liver . . | 2 | 1 | 1 | | | | | | | | 1 | | 1 | | | | | 3 |
| Congestion " . . | 1 | 1 | 2 | | | | | | | | | | | | | | | 2 |
| Consumption of Bowels | 4 | 2 | | 1 | | | | | 1 | | 2 | 1 | 1 | | | | | 6 |
| Colic . . . . | | 2 | 2 | | | | | | | | | | | | | | | 2 |
| Disease of Bowels . | | 1 | 1 | | | | | | | | | | | | | | | 1 |
| " Liver . . | 1 | 2 | | | | | | | | 1 | 1 | 1 | | | | | | 3 |
| " Stomach . | 1 | 3 | | | | | | | 2 | 1 | | | | 1 | | | | 4 |
| Dropsy of Abdomen . | 2 | 3 | | | | | | | 2 | | | 2 | | 1 | | | | 5 |
| Enlargement of Liver . | 3 | | 1 | | | | | | | | 1 | 1 | | | | | | 3 |
| Gangrene of Stomach . | | 1 | | | 1 | | | | | | | | | | | | | 1 |
| Hemorrhage of Bowels | 1 | | | | | | | | | | 1 | | | | | | | 1 |
| " Stomach | 2 | 2 | | | | | | | 1 | | 1 | 1 | | 1 | | | | 4 |
| Ileus . . . . . | | 1 | | 1 | | | | | | | | | | | | | | 1 |
| Induration of Liver . | | 1 | | | | | | | 1 | | | | | | | | | 1 |
| Inflammation of Liver . | 6 | 9 | | 2 | 2 | | | | 2 | 1 | 2 | 5 | 1 | | | | | 15 |
| " Stom'h & Bow'ls | 46 | 36 | 29 | 13 | 8 | 3 | 2 | | 7 | 4 | 5 | 4 | 1 | 3 | 3 | | | 82 |
| " Peritoneum . | 7 | 6 | 2 | | 1 | | 1 | | 3 | 2 | 2 | | 1 | | | 1 | | 13 |
| Jaundice . . . . | 4 | 3 | | 1 | 1 | | | | | 3 | 2 | | | | | | | 7 |
| Obstruction of Bowels . | 3 | 1 | 2 | | | | | | | 1 | 1 | | | | | | | 4 |
| Perforation of Intestines | 1 | | | | | | | | 1 | | | | | | | | | 1 |
| Scirrhus Stomach . | I | | | | | | | | | | | 1 | | | | | | 1 |
| Sore Mouth . . | 1 | | 1 | | | | | | | | | | | | | | | 1 |
| Teething . . . | 3 | 3 | 2 | 3 | 1 | | | | | | | | | | | | | 6 |
| Ulceration of Bowels . | 2 | 1 | | | | 1 | 1 | | | 1 | | | | | | | | 3 |
| " Stomach . | 1 | | | | | | | | | 1 | | | | | | | | 1 |
| " Stom'h & Bow'ls | 1 | 2 | | | 1 | | | | | | | 1 | 1 | | | | | 3 |
| | 94 | 87 | 46 | 22 | 16 | 4 | 4 | | 20 | 15 | 20 | 18 | 6 | 6 | 3 | 1 | | 118 |

### 7. *The Urinary Organs.*

| | Male. | Female. | Under 1 yr. | 1 to 2. | 2 to 5. | 5 to 10. | 10 to 15. | 15 to 20. | 20 to 30. | 30 to 40. | 40 to 50. | 50 to 60. | 60 to 70. | 70 to 80. | 80 to 90. | 90 to 100. | 100 to 110. | Total. |
|---|---|---|---|---|---|---|---|---|---|---|---|---|---|---|---|---|---|---|
| Diabetes . . | | 1 | | | | | | | | | | 1 | | | | | | 1 |
| Disease of Bladder . | 1 | | | | | | | | 1 | | | | | | | | | 1 |
| " of Kidneys . | 2 | | | | | | | | | | 1 | | 1 | | | | | 2 |
| Inflam. of Kidneys . | | 2 | 1 | | | | | 1 | | | | | | | | | | 2 |
| " of Bladder . | 1 | 1 | | | | | | | 1 | | | | | 1 | | | | 2 |
| Retention of Urine . | 1 | | 1 | | | | | | | | | | | | | | | 1 |
| | 5 | 4 | 2 | | | | | 1 | 2 | | 1 | 1 | 1 | 1 | | | | 9 |

### 8. *Organs of Generation.*

| | Male. | Female. | Under 1 yr. | 1 to 2. | 2 to 5. | 5 to 10. | 10 to 15. | 15 to 20. | 20 to 30. | 30 to 40. | 40 to 50. | 50 to 60. | 60 to 70. | 70 to 80. | 80 to 90. | 90 to 100. | 100 to 110. | Total. |
|---|---|---|---|---|---|---|---|---|---|---|---|---|---|---|---|---|---|---|
| Amenorrhœa . . | | 1 | | | | | | 1 | | | | | | | | | | 1 |
| Cancer of Uterus . . | | 3 | | | | | | | | | 2 | | 1 | | | | | 3 |
| Child Bed . . . | | 4 | | | | | | | 2 | 2 | | | | | | | | 4 |
| Chlorosis . . . | | 1 | | | | | | | 1 | | | | | | | | | 1 |
| Disease of Uterus . | | 1 | | | | | | | 1 | | | | | | | | | 1 |
| Hemorrhage " . | | 4 | | | | | | | 2 | 2 | | | | | | | | 4 |
| Inflammation " . | | 1 | | | | | | | | 1 | | | | | | | | 1 |
| Inverted " . | | 1 | | | | | | | | 1 | | | | | | | | 1 |
| Phlegmasia Dolens . | | 1 | | | | | | | | | 1 | | | | | | | 1 |
| Puerperal Convulsions | | 4 | | | | | | 1 | 2 | 1 | | | | | | | | 4 |
| " Fever . | | 8 | | | | | | | 6 | 1 | 1 | | | | | | | 8 |
| " Mania . | | 1 | | | | | | | 1 | | | | | | | | | 1 |
| Rupture of Uterus . | | 2 | | | | | | | 2 | | | | | | | | | 2 |
| | | 32 | | | | | | 2 | 17 | 8 | 4 | | 1 | | | | | 32 |

### 9. *Organs of Locomotion.*

| | Male. | Female. | Under 1 yr. | 1 to 2. | 2 to 5. | 5 to 10. | 10 to 15. | 15 to 20. | 20 to 30. | 30 to 40. | 40 to 50. | 50 to 60. | 60 to 70. | 70 to 80. | 80 to 90. | 90 to 100. | 100 to 110. | Total. |
|---|---|---|---|---|---|---|---|---|---|---|---|---|---|---|---|---|---|---|
| Caries of Vertebra . | 1 | | | | | | | | | | | | 1 | | | | | 1 |
| Disease of Hip . . | 2 | | | | | | | | 1 | 1 | | | | | | | | 2 |
| " of Spine . . | | 1 | | | | | 1 | | | | | | | | | | | 1 |
| Rheumatism . . | 1 | | | | | | | | 1 | | | | | | | | | 1 |
| | 4 | 1 | | | | | 1 | | 2 | 1 | | | 1 | | | | | 5 |

### 10. *The Integumentary System.*

| | Male. | Female. | Under 1 yr. | 1 to 2. | 2 to 5. | 5 to 10. | 10 to 15. | 15 to 20. | 20 to 30. | 30 to 40. | 40 to 50. | 50 to 60. | 60 to 70. | 70 to 80. | 80 to 90. | 90 to 100. | 100 to 110. | Total. |
|---|---|---|---|---|---|---|---|---|---|---|---|---|---|---|---|---|---|---|
| Purpura Hemorrhagica | 6 | 2 | 1 | 2 | 1 | | 1 | 1 | 1 | | 1 | | | | | | | 8 |

### 11. *Old Age.*

| | | | | | | | | | | | | | | | | | | |
|---|---|---|---|---|---|---|---|---|---|---|---|---|---|---|---|---|---|---|
| Old Age . . . . | 8 | 22 | | | | | | | | | | | 3 | 9 | 13 | 4 | 1 | 30 |

12. *From External Causes.*

| | Male. | Female. | Under 1 yr. | 1 to 2. | 2 to 5. | 5 to 10. | 10 to 15. | 15 to 20. | 20 to 30. | 30 to 40. | 40 to 50. | 50 to 60. | 60 to 70. | 70 to 80. | 80 to 90. | 90 to 100. | 100 to 110. | Total. |
|---|---|---|---|---|---|---|---|---|---|---|---|---|---|---|---|---|---|---|
| Asphyxia . . . | 7 | 4 | 10 | 1 | | | | | | | | | | | | | | 11 |
| Burns and Scalds . . | 2 | 5 | | 2 | 1 | 1 | | | 1 | | 2 | | | | | | | 7 |
| Casualties . . . | 20 | 4 | 3 | | 3 | 3 | 1 | 2 | 4 | 1 | 4 | 2 | 1 | | | | | 24 |
| Drowned . . . | 40 | 6 | | | | 6 | 4 | 3 | 10 | 14 | 6 | 3 | | | | | | 46 |
| Fracture of Leg, . . | 1 | | | | | | | 1 | | | | | | | | | | 1 |
| " of Pelvis, . | 1 | | | | | | | | 1 | | | | | | | | | 1 |
| " of Skull, . | 1 | | | | | | | | | 1 | | | | | | | | 1 |
| " of Spine . | 1 | | | | | | | 1 | | | | | | | | | | 1 |
| Gun Shot Wound, . | 2 | | | | | | 1 | | 1 | | | | | | | | | 2 |
| Hydrophobia, . . | | 1 | | | | | | | 1 | | | | | | | | | 1 |
| Intemperance . . | 10 | 10 | | | | | | | 5 | 6 | 5 | 4 | | | | | | 20 |
| Poisoning . . . | 1 | 2 | 1 | | 1 | | | 1 | | | | | | | | | | 3 |
| Suffocation . . . | | 2 | 1 | 1 | | | | | | | | | | | | | | 2 |
| Suicide . . . | 1 | 3 | | | | | | | 3 | 1 | | | | | | | | 4 |
| | 87 | 37 | 15 | 4 | 5 | 10 | 6 | 8 | 26 | 23 | 17 | 9 | 1 | | | | | 124 |
| Still Born . . . | 66 | 71 | 137 | | | | | | | | | | | | | | | 130 |
| Unknown . . . | 37 | 23 | 28 | 1 | 3 | | | | 4 | 10 | 6 | 7 | 1 | | | | | 67 |

### TABLE NO. 3.

*Deaths for the Third Quarter of* 1853, *at fifteen distinct periods of life.*

| | |
|---|---|
| Under 1 year, . . . . . . | 794 |
| 1 to 2 . . . . . . . | 363 |
| 2 to 5 . . . . . . . | 215 |
| 5 to 10 . . . . . . . | 90 |
| 10 to 15 . . . . . . . | 47 |
| 15 to 20 . . . . . . . | 97 |
| 20 to 30 . . . . . . . | 342 |
| 30 to 40 . . . . . . . | 276 |
| 40 to 50 . . . . . . . | 215 |
| 50 to 60 . . . . . | 151 |
| 60 to 70 . . . . . . . | 112 |
| 70 to 80 . . . . . . . | 70 |
| 80 to 90 . . . . . . . | 35 |
| 90 to 100 . . . . . . | 10 |
| 100 to 110 . . . . . . . | 1 |
| | 2818 |
| Still Born . . . . . . . . | 137 |
| Total, | 2955 |

Embraced within the foregoing tables, were 221 from the Blockley Alms House, 175 persons of color, and 35 from the country, as follows:

| | July. | August. | September. | Total. |
|---|---|---|---|---|
| Almshouse, . . | 57 | 93 | 71 | 221 |
| Blacks, . . . | 59 | 66 | 50 | 175 |
| Country, . . | 13 | 17 | 5 | 35 |
| Total, | 129 | 176 | 126 | 431 |

